# Supplementary material for: Vulnerability Assessment of Six Endemic Tibetan-Himalayan Plants Under Climate Change and Human Activities
Source: Plants (Basel). 2025 Aug 5;14(15):2424. doi: 10.3390/plants14152424 (PMC12349231; doi:10.3390/plants14152424)
Supplement: Supplementary file 1 [file plants-14-02424-s001.zip › plants-3719778-supplementary.pdf]

Table S1. Number of cleaned occurrence records and assigned threat category from the Red List of Chinese Plants for six plant species found on the Tibetan-Himalayan region.

Species	Occurrence records	Red List Category
S. kawaguchii	34	LC
A. erecta	99	LC
C. microphyllus	21	LC
S. purpurea	102	NT
I. younghusbandii	42	NT
A. xylorhiza	65	NT

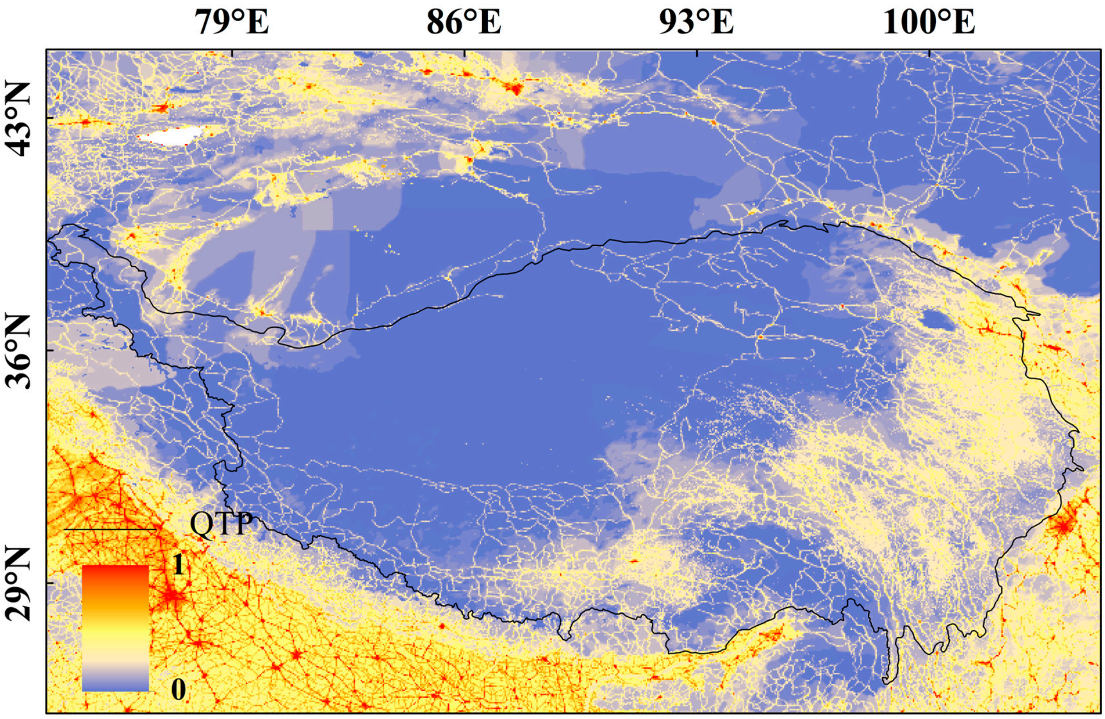

Figure S1. Human footprint maps of the Tibetan-Himalayan region normalized to within 0 to 1

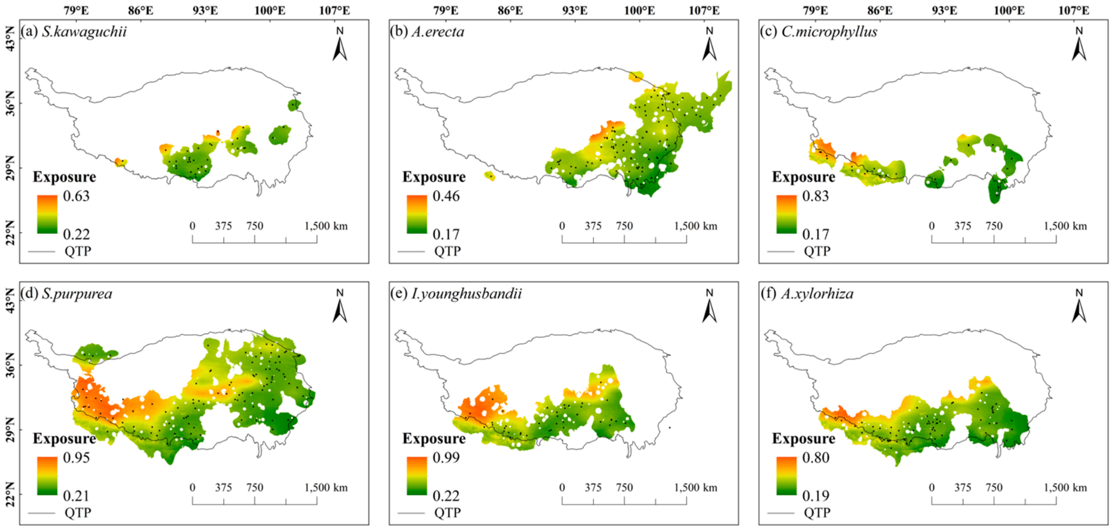

Figure S2. Exposure of six plant species on the Tibetan-Himalayan region under future climate change

scenarios (Shared Socio-economic Pathways; SSP5-8.5). The black dots in the figure represent the distribution of sample recording sites for the six plant species.

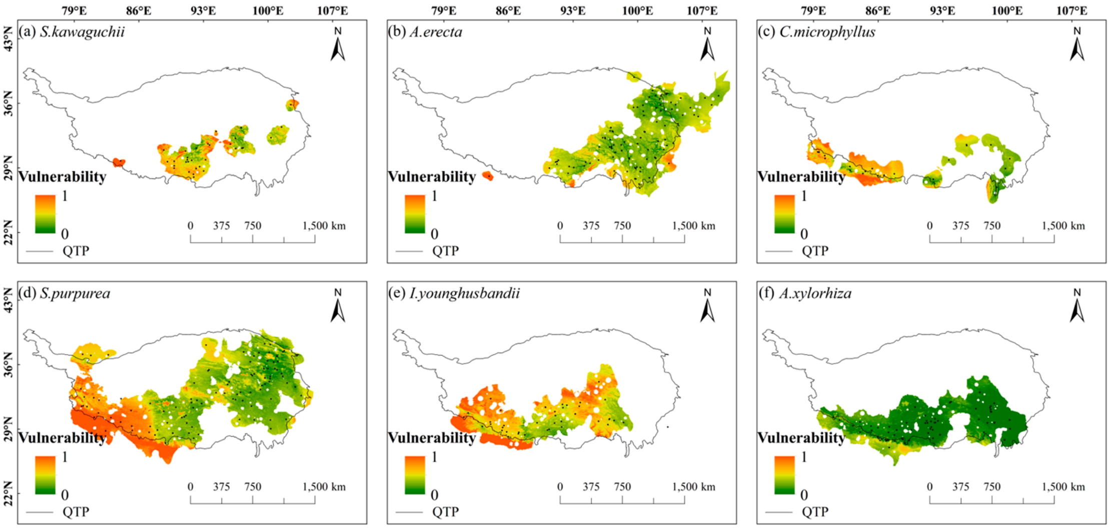

Figure S3. Vulnerability of six plant species on the Tibetan-Himalayan region under future climate change scenarios (Shared Socio-economic Pathways; SSP5-8.5). The black dots in the figure represent the distribution of sample recording sites for the six plant species.

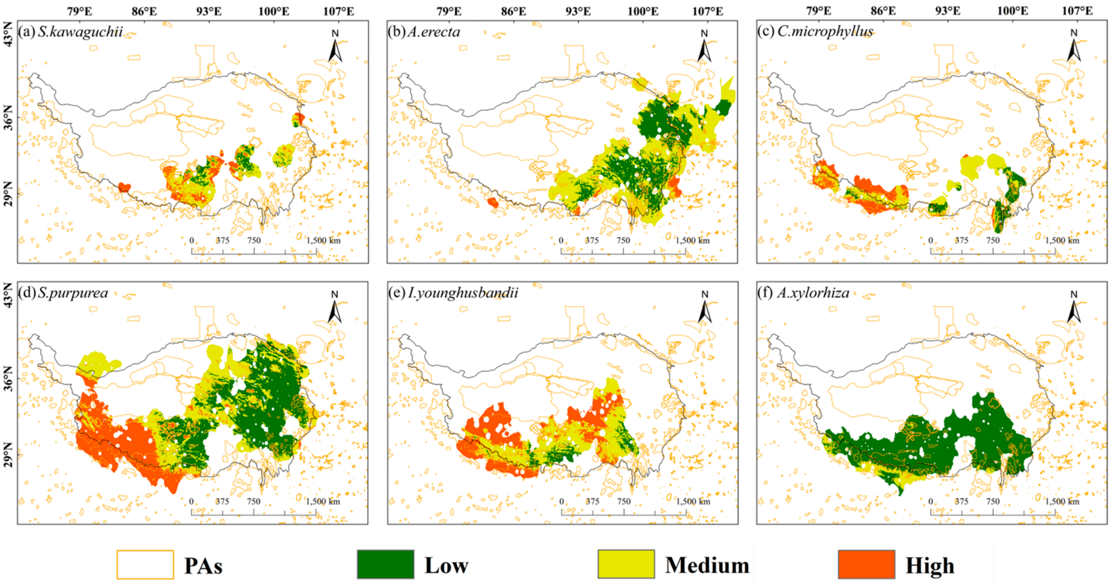

Figure S4. Under a future climate change scenario (Shared Socio-economic Pathways; SSP5-8.5), the vulnerability of six plant species on the Tibetan-Himalayan region was classified as low, medium, or high, and visualized through a color gradient: dark green for low vulnerability, light green for medium vulnerability, and red for high vulnerability. In addition, the areas marked with yellow outlines in the figure indicate the extent of protected areas, which were used to analyze the impact of protected areas on species vulnerability.

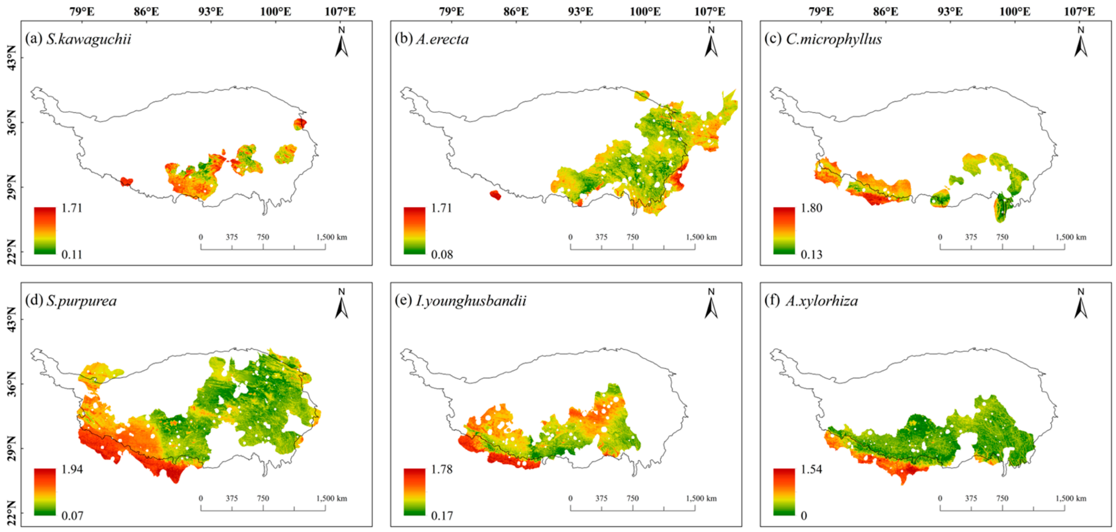

Figure S5. Vulnerability of six plant species in the Tibetan-Himalayan region under future climate change scenarios (Shared Socio-economic Pathways；SSP5-8.5) coupled with human footprints.
